# How Safety Culture Surveys Influence the Quality and Safety of Healthcare Organisations

**DOI:** 10.7759/cureus.44603

**Published:** 2023-09-03

**Authors:** Rob M Bethune, Sue Ball, Natasha Doran, Michael Harris, Antionieta Medina-Lara, Mauro Fornasiero, Matt Hill, Iain Lang, Judith McGregor-Harper, Rod Sheaff

**Affiliations:** 1 Colorectal Surgery, Royal Exeter University Healthcare NHS Foundation Trust, Exeter, GBR; 2 National Institute for Health and Care Research (NIHR) Applied Research Collaboration, South West Peninsula (PenARC), University of Exeter Medical School, Exeter, GBR; 3 College of Medicine and Health, University of Exeter Medical School, Exeter, GBR; 4 Institute of Primary Health Care (BIHAM), University of Bern, Bern, CHE; 5 Department of Public Health and Sports Science, Public Health Economics Group, University of Exeter Medical School, Exeter, GBR; 6 School of Law and Criminology, University of Plymouth, Plymouth, GBR; 7 Anaesthesia, University Hospital Plymouth NHS Trust, Plymouth, GBR; 8 Department of Health and Community Sciences, University of Exeter Medical School, Exeter, GBR; 9 Health Policy, Care Quality Commission, London, GBR; 10 Faculty of Medicine and Dentistry, University of Plymouth, Plymouth, GBR

**Keywords:** qualitative semi-structured interviews, qualitative studies, qualitative, health policy making, surveys, safety, culture

## Abstract

Objectives

Safety culture surveys have been widely used in healthcare for more than two decades predominantly as a tool for measuring the level of safety culture (as defined as the beliefs and attitudes that staff express about how their organisation ought to work and how it does in fact work). However, there is the potential for the survey process itself to influence the safety culture and working practices in departments and organisations. The objective of this study was to identify the mechanism by which these changes might occur.

Design, setting and participants

Mixed methods combining qualitative semi-structured interviews and quantitative scores from patient safety surveys.

This evaluation was conducted across general practice, community and acute hospitals in two NHS regions in England; South West and Greater Manchester. The study was undertaken between 2015 and 2018 during the implementation of a series of Patient Safety Collaboratives. Safety, Communication, Operational Reliability, and Engagement (SCORE) surveys were administered in 15 units, followed by a staff debriefing and a second SCORE survey. Semi-structured interviews were conducted with clinicians (n=61). Results from the first and second surveys were compared in order to test for differences in responses. Sixty-one semi-structured interviews were conducted across participating units and thematically analysed.

Analysis and results

Results from the first and second surveys were compared using chi-squared and Fisher’s exact tests. Sixty-one semi-structured interviews were conducted across participating units and thematically analysed.

There was little change in responses between the first and second SCORE surveys. Within general practice there was some improvement in responses in three survey domains; however, these differences were not conclusive. The qualitative interview data demonstrated a beneficial effect on safety culture. Staff stated that the survey debriefings created a new safe space where problems could be discussed and improvement plans created.

Conclusions

Safety culture surveys can improve safety culture within departments if they are followed by a process that includes debriefing the staff and working with them to develop improvement plans.

## Introduction

Safety culture surveys are widely used to measure safety culture within healthcare organisations. Their use is based on the presumption that safety culture is correlated to clinical outcomes, an assumption that some (but not all) studies in healthcare support [1-6]. Studies in other industries (e.g. aviation and oil exploration) also report correlations between safety culture scores and worker safety, satisfaction, stress and accident levels [7,8]. Some published papers define safety itself as the profile of scores in these surveys [9-11]. 

For the purpose of this paper, an organisation’s safety culture is defined as the beliefs and attitudes that staff express about how their organisation ought to work and how it does in fact work. Similarly, safety climate is defined as the beliefs and attitudes staff have about how their frontline units and clinical teams ought to work safely and what actually happens. Therefore, safety culture surveys are really a measure of local workplace climate; however, as much of the published literature does not reliably distinguish between culture and climate and mostly uses the term ‘safety culture’ to refer to the safety climate within individual frontline units, we have kept that usage for this study.

A frontline unit or department is the basic work-team of a health organisation, e.g. a pharmacy, an emergency department or a general practice. Existing studies, and the safety management practices based upon them, tend to assume that the results of the surveys can be used to identify organisations and work-teams within them where the safety culture is deficient; and that suitable interventions by the organisations’ managers and clinical leaders can change that culture and by doing so improve the clinical outcomes for patients [12-14]. Multiple studies have assumed that any consequent improvement in safety culture survey scores is a proxy, or even a predictor, of higher quality of care [2-4,7,10,15-17]. The evidence for this assumption is varied; one study has shown that improving culture scores improves the quality of care [18]; whereas others show that when those scores improve, there is no change in clinical outcomes and that when clinical outcomes for patients improve the safety culture score had not changed [4,16,19]. The reasons and mechanisms explaining why changing safety culture affects measurable clinical outcomes in some settings and not in others are not known. However, some studies have conjectured that safety culture surveys themselves can improve the culture rather than being just a measurement tool [20,21]. A possible mechanism could be that the safety culture survey results are used to facilitate post-survey workshops or debriefing sessions between management and frontline staff to develop activities that address potential cultural weaknesses [22]. Other possibilities (compatible with the first) are the Hawthorne effect (the very act of observing individuals changes their productivity or other behaviours) [23,24] or the creation of a climate in which individuals feel psychologically safer to take the risks of raising potentially contentious issues and trying out new working practices [25].

Of the many patient safety culture survey instruments [26-30], the Safety Attitude Questionnaire is the original and was adapted from an aviation culture survey [27]. For this study we used the updated and validated version: the Safety, Communication, Operational Reliability, and Engagement (SCORE) survey [5].

In response to the Francis Inquiry into the systematic poor quality of care given at Mid-Staffordshire NHS Trust, the English NHS implemented 15 Patient Safety Collaboratives (PSCs) in 2015. The policy documents and guidance establishing PSCs emphasised changing safety culture as a means of improving patient safety. As part of this implementation, safety culture surveys were conducted both as a measurement and, as described in more detail below, as an intervention tool.

The two objectives of this study were to assess the influence of the SCORE survey on the working practices of frontline units and to analyse the numerical results of these surveys.

## Materials and methods

Setting and design

The Patient Safety Collaboration Evaluation Study (PiSCES) was funded by the National Institute for Health and Care Research (NIHR) to evaluate the effect of the PSCs. This included administration of SCORE surveys before and after the implementation of the PSCs with the intention of observing any numerical change in the survey results and a qualitative evaluation of the effect of those safety culture surveys on staff and their working practices.

The study was conducted across 15 units in two English NHS regions, the South West and Greater Manchester, which between them cover a population of around 8 million.

The SCORE survey administration

We used the following seven domains of the SCORE survey: learning environment (six questions), teamwork climate (seven questions), safety climate (seven questions), burnout climate (five questions) and personal burnout (five questions), perceptions of local leadership (seven questions) and work-life balance (eight questions). Table 1 describes each of these domains. The survey also collects free-text comments.

**Table 1:** SCORE survey domains SCORE: Safety, Communication, Operational Reliability, and Engagement [5]

Domain	Description
Learning environment	How well staff can learn and improve from previous performance ‘The learning environment allows us to pause and reflect on what we do well’
Teamwork climate	How the team works and communicates together ‘The people here work well together as a well-coordinated team’
Safety climate	Staff perception of how safe the unit is ‘I would feel safe being treated here’
Burnout climate	How others perceive individual burnout ‘In this work setting, people are burnt out from their work’
Personal burnout	How individuals perceive their own level of burnout ‘During the past week how often did you have difficulty sleeping?’
Perceptions of local leadership	How well leadership; provides useful feedback, creates an open culture and resolves disputes ‘The values of our leaders are the same values that people in this work-setting think are important’
Work-life balance	The impact of work on personal life ‘During the past week, how often did you arrive home late from work’

Each question has possible responses on a Likert scale from 1 (strongly disagree) to 5 (strongly agree) for all the domains except for the work-life balance questions which are answered on a scale from 1 to 4: rarely or none of the time (less than one day); some or a little of the time (one to two days); occasionally or a moderate amount of time (three to four days); all of the time (five to seven days).

The survey and debriefing process was provided free of charge for organisations within the two study regions. Fifteen units agreed to take part in the survey including 10 general practices, three hospital departments (emergency, medical admission unit and maternity), one community mental health pharmacy and one social care organisation.

Response rates were fed back to the units’ leaders on a weekly basis. After six weeks or when the response rate reached 60%, the survey was closed. The responses were summarised in a report and shared with the participating units.

The survey was repeated in the same units between 12 and 18 months after the initial survey.

Survey debriefing

The units’ senior leadership were debriefed first by trained external facilitators and the remaining staff were debriefed in groups with an emphasis on ensuring that staff felt psychologically secure to openly discuss the results. The debriefing process was designed to be an opportunity for the team to; celebrate their good work, explore why they had answered questions in a certain way, identify the different perceptions within the groups and identify any opportunities for improvement.

Quantitative analyses of the SCORE results

Individuals’ scores for the SCORE survey, for the six domains with 5-point Likert scales, were calculated as follows: the response to each question within the domain was converted to 0-100 by subtracting 1 from the numeric response and then multiplying by 25 (inverting any negatively worded questions in the teamwork and safety climate domains). An individual’s domain score was calculated as the mean of the 0-100 scaled question responses, based on all the questions that the individual answered within that domain. Thus if an individual has a domain score of 100 they have responded ‘agree strongly’ to all questions that they have answered within that domain; 75 indicates that their mean response was ‘agree slightly’; 50 indicates that their mean response was ‘neutral’; 25 indicates that their mean response was ‘disagree slightly’, and 0 means that all responses were ‘disagree strongly’. Individuals’ scores for the seventh (work-life balance) domain were the means of the numeric responses to each question within that domain.

Individuals’ domain scores were dichotomised into positive or negative responses as follows: for learning environment, local leadership, safety climate and teamwork domains, scores of 75 or higher were positive and scores of less than 75 were negative. For burnout climate and personal burnout domains, scores of less than 50 were positive and scores of 50 or higher were negative. For work-life balance, scores of less than 2.1 were positive and scores of 2.1 or higher were negative. 

Scores from the 10 general practices were combined into a single unit of general practices for analyses. Individuals’ domain scores were summarised in terms of the number and percentage of positive responses and were compared between the first and second surveys using chi-squared and Fisher’s exact tests. All analyses were carried out using R statistical software (version 3.5.1; R Foundation for Statistical Computing, Vienna, Austria).

Qualitative evaluation of survey effects

To discover how units responded to the survey and its results, we carried out 61 interviews across participating units and included three organisational levels of the NHS. These were PSC leads (N = 16); patient safety leads within provider organisations (N = 14); and frontline staff (N = 31). All PSC leads took part in our study. Selection of other interviewees was stratified (i.e. purposively selected) to provide a cross-section of managerial and clinical roles.

All interviewees were contacted by e-mail and returned completed and signed consent forms before taking part in the study. Interviews were carried out by telephone and lasted between 25 and 110 minutes. Interviews used a semi-structured interview schedule which was designed by the research team. Interviews were audio recorded, transcribed and anonymised. Thematic analysis techniques were then used to generate a description both within and across the dataset [31].

Patient and public involvement (PPI)

Patient involvement in this project ensured we dealt with questions that make sense to patients and members of the public. Two expert patients were involved in the preparation of the research protocol. Another patient representative joined as a member of the steering committee. At the end of the project we met a wider group of patient representatives who advised on how to present, interpret and disseminate the findings to maximise their relevance and usefulness to patients. We sought and received expert PPI advice from our NIHR CLAHR partners (CLAHRC for the South West Peninsula [PenCLAHRC]).

Ethical approval was obtained from the NHS Research Ethics Service (15-NI-0235).

## Results

SCORE survey results

The number of responses to each of the survey domains varied across units from 13 to 379 and the percentage responses ranged from 57% to 81%.

Table 2 summarises the SCORE survey results for the 10 combined general practices and the remaining five units, for each of the seven domains and compares the percentage of positive responses between first and second surveys.

**Table 2:** SCORE survey results p values are from chi-squared (for general practices, hospital emergency department (ED), medical assessment unit (MAU), social care and hospital maternity) or Fisher’s exact tests (for pharmacy) comparing the percentage of positive responses between first and second surveys. SCORE: Safety, Communication, Operational Reliability, and Engagement

Unit		General practices			Hospital ED			Hospital MAU			Mental Health Pharmacy			Social Care Organisation			Hospital Maternity		
Domain	Survey number	n (%) response	n (%) positive	p	n (%) response	n (%) positive	p	n (%) response	n (%) positive	p	n (%) response	n (%) positive	p	n (%) response	n (%) positive	p	n (%) response	n (%) positive	p
Learning environment	1	341 (66)	194 (56.9)	0.02	111 (58)	64 (57.7)	0.4	83 (58)	39 (47.0)	0.7	13 (72)	9 (69.2)	0.3	66 (76)	57 (86.4)	0.2	97 (66)	67 (69.1)	0.5
	2	376 (71)	247 (65.7)		150 (66)	77 (51.3)		96 (61)	49 (51.0)		13 (81)	12 (92.3)		78 (76)	73 (93.6)		102 (63)	65 (63.7)	
Local leadership	1	340 (66)	136 (40.0)	0.002	110 (57)	41 (37.3)	0.3	83 (58)	30 (36.1)	0.4	13 (72)	12 (92.3)	0.2	66 (76)	50 (75.8)	0.7	96 (65)	31 (32.3)	1
	2	377 (71)	195 (51.7)		150 (66)	45 (30.0)		95 (61)	41 (43.2)		13 (81)	8 (61.5)		78 (76)	56 (71.8)		102 (63)	33 (32.4)	
Burnout climate	1	342 (67)	100 (29.2)	<0.001	113 (59)	16 (14.2)	0.5	84 (58)	9 (10.7)	0.7	13 (72)	2 (15.4)	0.6	66 (76)	30 (45.5)	0.7	97 (66)	32 (33.0)	0.03
	2	379 (72)	162 (42.7)		151 (67)	16 (10.6)		96 (61)	13 (13.5)		13 (81)	4 (30.8)		78 (76)	39 (50.0)		102 (63)	19 (18.6)	
Personal burnout	1	343 (67)	170 (49.6)	0.07	113 (59)	38 (33.6)	0.8	85 (59)	26 (30.6)	0.4	13 (72)	5 (38.5)	1	66 (76)	56 (84.8)	0.07	97 (66)	50 (51.5)	0.8
	2	379 (72)	214 (56.5)		151 (67)	47 (31.1)		97 (62)	37 (38.1)		13 (81)	6 (46.2)		78 (76)	55 (70.5)		102 (63)	50 (49.0)	
Teamwork	1	341 (66)	168 (49.3)	0.1	113 (59)	39 (34.5)	0.1	84 (58)	29 (34.5)	0.8	13 (72)	2 (15.4)	0.1	66 (76)	48 (72.7)	0.3	97 (66)	46 (47.4)	0.3
	2	379 (72)	209 (55.1)		150 (66)	38 (25.3)		97 (62)	31 (32.0)		13 (81)	7 (53.8)		78 (76)	49 (62.8)		102 (63)	57 (55.9)	
Safety climate	1	342 (67)	200 (58.5)	0.2	113 (59)	41 (36.3)	0.5	84 (58)	22 (26.2)	0.09	13 (72)	7 (53.8)	0.7	66 (76)	56 (84.8)	0.8	97 (66)	46 (47.4)	0.3
	2	378 (71)	238 (63.0)		151 (67)	47 (31.1)		97 (62)	38 (39.2)		13 (81)	9 (69.2)		78 (76)	64 (82.1)		102 (63)	57 (55.9)	
Work-life balance	1	333 (65)	235 (70.6)	1	112 (58)	60 (53.6)	0.9	82 (57)	38 (46.3)	0.7	13 (72)	9 (69.2)	1	64 (74)	45 (70.3)	0.2	95 (65)	57 (60.0)	0.6
	2	366 (69)	259 (70.8)		149 (66)	82 (55.0)		95 (61)	48 (50.5)		13 (81)	10 (76.9)		77 (75)	45 (58.4)		101 (62)	65 (64.4)	

Among general practices there was some indication of an increase in the percentage of positive responses from the first to second survey in the learning environment, local leadership and burnout climate domains. However, as many statistical tests were performed these results should be interpreted with caution. There was little evidence of a difference in the percentage of positive responses between the first and second surveys within general practices for the other domains, or for any of the remaining units on any of the seven domains.

As the organisational setting may influence individuals’ and units’ responses to the survey, the qualitative findings are presented by clinical setting. 

General practice

During the two years of the evaluation, policy, regulatory and organisational changes were continuous. In one specific case the collapse and subsequent merging of four general practices gave the merged practice the opportunity to standardise pathways in several areas including patient demand and flow, prescribing, infection control and nursing registrations. At the point of interview this practice was nine months after the first SCORE survey which had been carried out while staff were adapting to their new organisation.

Following the practice merger, patient demand, communication across teams, and the role leadership has within the teams were identified as the biggest challenges. Our informants reported that the initial survey results and debrief had proved effective and timely in enabling changes to be identified in these areas and “to identify groups that were in particular need of attention” (Practice partner PSCOL4). Following the debrief session the participants had agreed to: undertake more regular appraisals; take the time to listen to staff; ensure all staff knew who their line managers were and felt comfortable in approaching them should any problems arise; and bring in new measurements of process and patient outcomes and ensure staff were confident and fully trained in their use:

“People are still going through that change cycle and some have got there. All the GPs on the first survey scored brilliantly because […] they each had a project that they were driving along and they were fully engaged. We’ve slowly brought the rest of the teams along by trying to explain why and what we’re doing and they can actually be part of it and what would they like to do to make it better” (PSCOL4 Practice partner).

In this general practice the SCORE survey proved a useful mechanism to support organisational change partly because it coincided with the practice merger and complete redesign of their service.

In another general practice, staff had taken part in the SCORE survey and completed two rounds and debriefs by the time of interview. According to this practice manager:

“The staff got a chance to really think about the structure of the team here and with the amount of questions that they had to answer, I think it really delved deep into what people think about the communication, the management style, the leadership and the teams and how they integrate and engage with each other.” (PSCOL12 Practice manager).

Following the first debrief, this practice implemented a number of changes to improve communication among the senior team, such as introducing a handover booklet to record and date information for colleagues to check daily; and to improve patient safety by applying a more systematic and rigorous approach to any significant events through recording data more effectively and applying a greater depth of analysis, either through a written report or through a meeting with recorded minutes and a clear action plan.

These changes demonstrate a ‘virtuous circle’ of survey-debrief-improvement followed by a repeat survey. The practice manager was keen to ensure these positive results continued to improve:

“To keep that open level of communication and the no blame culture going and the sense of one team going, because I think that is very important here, and it makes a huge difference.” (PSCOL12 Practice manager).

Other general practices developed improved communication with line managers and more regular meetings for feedback; practice partners having an “open door policy”, as well as the “up-skilling” of staff, so that they were better able to support colleagues where necessary, including receptionists being trained to prioritise the needs of patients in order to improve safety.

No informant in any of these general practices reported any adverse consequences from the survey or post-survey debriefing.

Community mental health pharmacy

In the community mental health pharmacy, the first SCORE survey had suggested that the clinical team was high-performing, productive and delivering care to a high standard, but nevertheless underlying problems with communication, teamwork and burnout were all negatively affecting staff well-being:

“It [SCORE survey] showed us that yeah, actually we had all the great ingredients to be this perfect team that everyone perceived us as, but there were some key hotspots in terms of our communications, teamwork, feeling of our own burnout and workload, and the burnout culture within the team and the organisation, that was possibly kind of having a negative impact on the overall feeling about what it felt like to work in our team” (PSCOL14 Lead pharmacist).

One other participant from this team described “unhealthy competition” across the team with a “martyr approach” to being busy:

“Like any NHS team at the moment, you can't control demand, so workload is quite out there and so there is this sort of martyr sort of approach about who can be the busiest [which generates a] kind of unhealthy competition” (PSCFL22 Accountable Officer).

In this site, our informants stated that implementing the survey and having the results and debrief sessions facilitated externally, had a positive impact on the team:

“It [SCORE survey] really helped us to kind of have a very mature and objective conversation about how sometimes the actions and behaviour and interactions of other people that you witness can have a negative impact on you and, most importantly, your ability to do a good job” (PSCFL22 Accountable Officer).

Through offering a “safe space” and a “framework” to “depersonalise” and so broach those “difficult conversations,” (PSCFL22; PSCOL14) they viewed the SCORE survey as an ideal mechanism for illuminating problems and facilitating change.

A major problem with recruitment and retention as well as sickness absence was also reported within community mental health across this region with a sickness absence rate of 14%. However, after implementing these interpersonal behavioural changes at work, sickness absence had dropped to 0.6% by the time of the second survey.

Hospital departments

In the acute sector the survey provided the space and time for hospital staff to reflect upon their own attitudes as well as their colleagues’ perspectives:

“It [SCORE Survey] highlighted individuals’ perspectives that we had perhaps never even considered before and that [are] possibly very important in terms of delivering safe care to patients. So it highlighted the perspective of all different workers from the receptionist, three porters and all tiers of doctor and nurse and what they felt was important” (PSCFL1 Consultant ED).

This more inclusive approach whereby all staff levels and tiers from consultant through to porter were invited to take part in the debrief sessions, was corroborated by other members of the team:

“It [SCORE Survey] identified things that we’d never even thought about.” […] “It highlighted that actually, you know it’s just as bad for them [porters] as it is for anyone else, so they’re included if you have a de-brief, they always ask porters.” (PSCFL2 Nurse ED).

The SCORE survey showed that one of the main problems in the emergency department was burnout and the need for more staff to cope with overcrowding:

“It has been useful because the first time round it gave us a baseline picture of where we are as a department within an organisation […] it illustrated numerically what a burned out environment we are working in and how close to the edge quite a lot of our staff groups were.” (PSCFL1 Consultant ED).

In this ED, the percentage of positive responses to the burnout climate domain of the SCORE survey was 14% (16 out of 113) and 11% (16 out of 151) for the first and second surveys, respectively. These were some of the highest burnout scores in the study. Being able to demonstrate these results and explore the reasons for them led to better communication within teams and between frontline staff and senior management:

“We were able to demonstrate the key issues are staffing, space and flow of patients i.e. you know, we have a crowded department […] we were able to use the data and present it up the food chain to the executive directors. […] Just having that numeric data available has enabled us to get more staff which is helpful. But unfortunately crowding and space is still a massive issue.” (PSCFL1 Consultant ED).

In this case, hospital staff stated that implementing the SCORE survey had enhanced two-way communication across the hospital system:

“Because we’ve actually got evidence, it shows managers and you know people at a higher level that this has to change.” (PSCFL2 Nurse ED).

Following the SCORE survey and debrief sessions vital information was made available to senior management and senior executives who were equally keen to know the results. This in turn led to quicker decision-making and tangible improvements such as the rapid recruitment of more staff to help with overcrowding in the department. Additionally, the SCORE survey was reported to improve incident reporting and led to a series of safety days being set up following the results and debrief sessions. What is noticeable is that despite these positive actions, the numerical survey score in this department did not improve.

Though some informants across the sites felt it was “early days” in terms of determining the impact of the SCORE survey on patient care; many felt that through acknowledging staff well-being as a vital prerequisite for compassionate and high-quality care, improvement was already noticeable. Overall the reaction to the survey-debrief-improvement-survey circle was positive. However, one unit in the acute sector did struggle to get the required response rate, as it had just merged with another organisation resulting in leadership changes and a delay in the subsequent steps of the cycle (PSCFL10 Head of Nursing).

## Discussion

The qualitative data from this study show that a safety culture survey in conjunction with debriefing and consequent improvement planning can have a positive impact on the safety culture in the units completing the surveys. While this effect is seen in the qualitative data it is not reflected in the quantitative results of the survey.

The key part of the process appears to be the debriefing after the survey has been completed. Senior leaders ran sessions with frontline staff to present the results and then as a group to work on strategies to improve working practices and culture. The debriefing itself had multiple benefits; providing a safe space for feedback, legitimising the discussion of problems that had been previously ‘off-limits’ and acknowledging staff-wellbeing as a vital prerequisite for compassionate, high quality and safe patient care. Specific topics and factors were identified at the debriefings so that improvements could be tested to help; decrease sickness absence, reduce stress, improve communication and enhance teamwork. Besides these practical consequences our informants perceived that they were receiving greater mutual recognition of their work and greater managerial attention, consistent both with a Hawthorne effect and with increased psychological safety [23-25]. There appeared to be a ‘virtuous circle’ following the debriefing, staff became more engaged in developing their working environment which led to improvements in the culture which in turn allowed further improvements in working practices. Safety culture surveys have previously been used as a developmental tool; but as far as we are aware this study is the first to suggest the underlying mechanisms of how this process works [22,31,35].

The quantitative analysis of the 15 units who completed the survey cycle; first survey, debrief, improvement activity and then repeat survey showed little evidence of a change in the responses from first to second survey.

This leaves a paradox in that although the qualitative data suggests a positive impact on the safety culture this was not confirmed by the numerical data. There are several possible reasons for this; the time between each survey may have been too short, safety culture surveys may not have been measuring practically important aspects of safety culture or there may have been a change in the ‘mental set point’ of the staff within the unit: if an individual is working in a department where the safety culture improves their response to a question might not change as their threshold for what they consider safe working practice may also have increased.

Limitations

Departments self-selected to take part in the surveys. This could reduce the generalisability of the findings as volunteer units might be more open to the survey results and therefore any improvements relating to them. However defined, culture change within departments and organisations takes time and both the repeat surveys and interviews happened within 18 months of the first survey. Effects seen could develop further over time though equally the effects could reduce back to baseline after longer periods of time. A longer-term study to include ethnographic fieldwork would help establish whether safety culture changes because of the introduction of safer working practices, or whether an improvement in safety culture causes the development of safer working practices, or both (a ‘virtuous circle’). In two of the general practices we studied, the post-survey effects occurred in the contexts of a practice merger where the conditions were ripe for change, and in a practice that was already embracing innovation. Whether the same would happen in other general practice contexts remains unknown, although one study suggests that it might [22]. More generally workplace culture and climate may also change for other external reasons besides those considered here; additionally the sample sizes may have been too small to detect significant quantitative changes. 

## Conclusions

Both frontline staff and managers reported that using safety culture surveys as a developmental tool has a significant positive effect on their working patterns and safety culture. The surveys themselves do not create this effect, the key is a structured debrief of the staff with their managers after the survey has been run and using it as a starting point for open conversations. This then stimulates improvement activity completing the survey-debrief-improvement circle. 

The scores from the safety culture survey did not show significant change despite the positive qualitative findings. These findings emphasise the limited importance of the numerical results of the survey and support the use of the whole survey-debrief-improvement circle as a developmental tool for improving working practices and providing safer patient care.
